# Comparative History of the Diseases Transmissible from Domestic Animals to the Human Race

**Published:** 1842-01

**Authors:** 


### ART. II.

1. *Vergleichende Darstellung der von den Hausthieren auf Menschen übertragbaren Krankheiten—nach seiner von der medicinischen Facultät zu Berlin gekrönten Preisschrift bearbeitet von* JACOB LEVIN, Doctor der Medicin und Chirurgie.—*Berlin*, 1839. 8vo, pp. 292.

*Comparative History of the Diseases transmissible from Domestic Animals to the Human Race. Founded on a Prize Essay presented to the Faculty of Medicine of Berlin.* By JACOB LEVIN.—*Berlin*, 1839.

2. *De la Morve et du Farcin chez l'Homme.* Par M. RAYER. (*Mémoires de l'Académie Roy. de Médecine.*)—*Paris*, 1837. Tome VI. 4to.

*On Glanders and Farcy in the Human Subject.* By M. RAYER.

"THE deviation of man from the state in which he was originally placed by nature seems to have proved to him a prolific source of diseases From the love of splendour, from the indulgences of luxury, and from his fondness for amusement, he has familiarized himself with a great number of animals which may not originally have been intended for his associates." Such are the words with which the illustrious Jenner commences his Inquiry into the Causes and Effects of the Variolæ Vaccinæ, and taken in connexion with the subject of his investigation they seem to indicate a suspicion of the existence of numerous morbid poisons of animal origin. Such suspicion is very probably well founded; but this field of investigation has attracted few enquirers, and since the publication of Jenner's work the poison of glanders has alone been added to those previously known.

The diseases described by Dr. Levin are—glanders; malignant pustule; aphtha epizootica; cowpox; grease; itch (scabies ferina); and hydrophobia: with regard to each he describes the phenomena of the disease both in the animal in which it originates and in the human subject; its contagiousness and mode of transmission are next considered; its parallelism in the domestic animal and man is then discussed; and finally its prophylaxis and treatment are noticed. This arrangement is methodical, and we shall for the most part adhere to it in analyzing the work.

I. GLANDERS, (*Rotze.*) The horse, the ass, and the mule are the only animals in which, so far as we know, glanders is spontaneously generated. Indeed it does not seem to have been till recently suspected that any animals but those of this class were capable of contracting the disease, as in most works on veterinary medicine the malady is expressly stated to be peculiar to the solipedes or monodactyles.

Glanders assumes two forms, glanders commonly so called, and farcy. These varieties have been indeed considered as two distinct diseases; but we shall again examine this question, and first consider the symptoms and lesions of the various forms of the disease in the horse, that we may be enabled to compare it with the malady as it occurs in man.

Dr. Levin justly observes that different writers give very discordant accounts of the symptoms and morbid anatomy of glanders, and that it is consequently difficult to frame an unexceptionable description of the disease. His own history of the malady is extremely imperfect, as he appears to consider it as an uniformly chronic disease; his remarks on farcy, as unconnected with what is usually termed glanders, are also very unsatisfactory and incomplete. The truth is that glanders and farcy occur in both the acute and chronic form, thus constituting four varieties of the disease, each of which is very probably capable of producing a corresponding affection in the human subject. It consequently is necessary to advert to the leading characters of each of these varieties of the disease, as much light is thus thrown on the forms it assumes when transmitted to man.

*Chronic glanders* almost constantly commences with a nasal flux, usually from but one nostril; the discharge, at first aqueous and transparent, gradually assumes a yellow or greenish tinge, becomes purulent, sanguineous, and fetid, and at the same time acquires an adhesive consistence. The Schneiderian membrane is at first frequently preternaturally red, but subsequently becomes pale and flabby, and at length presents ulcers which have been usually compared to chancres; these ulcers extend slowly, and in their progress often produce caries of the subjacent bone. At a variable period from the commencement of the coryza, sometimes but very rarely before its appearance, a submaxillary lymphatic gland corresponding to the affected nostril becomes swollen, hard, and apparently adherent to the jaw. The general health may remain unaffected for a long period, even for years. Acute glanders may supervene on, or farcy may either precede or accompany the disease; but independently of these complications the animal ultimately becomes feeble, loses appetite and flesh, the respiration gets embarrassed, cough sets in, and death finally occurs amid symptoms resembling those of hectic fever. We have merely sketched the leading features of the disease as sufficient for the object we have in view. It is necessary, however, to describe the morbid appearances more minutely, and in so doing we shall avail ourselves of M. Rayer's memoir, which Dr. Levin does not appear to have consulted.

*The Schneiderian membrane* presents several alterations probably corresponding to different stages of the disease. Sometimes its surface is occupied by minute white solid granular elevations, containing neither pus nor tubercular matter; their number is very variable, and they may be discrete or grouped together, forming a rough elevated surface. In a more advanced stage they seem to coalesce and form smooth yellowish prominences, which at length become softened in the centre and ulcerate. The ulcers, when the disease has been truly chronic, are of a yellowish gray colour, without any surrounding vascularity; they seldom extend to the cartilage or bone, and their secretion somewhat resembles pus. The mucous membrane itself is yellowish and covered with thick, yellow, ropy mucus. The subjacent bones are sometimes thickened, porous, white, and friable; or may, on the contrary, be very hard but brittle. In the *larynx* and *trachea* ulcers similar to those above described were found by M. Rayer in two out of fifteen cases. In the *lungs* two lesions were constantly observed: 1st, small, hard, whitish cartilaginous granulations scattered on the surface or in the substance of the lung, but never to such an amount as to destroy its crepitation, or cause a portion of it to sink in water; and 2dly, appearances corresponding to the various stages of chronic circumscribed lobular pneumonia. M. Rayer found the submaxillary lymphatic glands enlarged, harder, and less vascular than natural. If, as often occurs, farcy coexists with glanders, the appearances proper to the former disease are of course present.

*Acute Glanders* is now pretty generally considered with M. Dupuy to be of two kinds, and as each may possibly be the type of a corresponding variety in man, we shall briefly describe their prominent characters.

A. *Pustular Glanders* consists in an acute inflammation of and pustular eruption on the Schneiderian membrane. The affection commences with constitutional disturbance and fever; the pituitary membrane is red, injected, and soon presents pustules with red bases and white summits; there is a sanious discharge from the nostrils, and the submaxillary glands become painful and swollen; in two or three days the pustules ulcerate and the nasal flux becomes more copious and adhesive; the posterior extremities, the sheath and nostrils swell, the respiration becomes extremely embarrassed; circumscribed, rounded tumours, which rapidly soften, frequently form in the skin, which is also occasionally but rarely the seat of a pustular eruption in certain situations, especially about the sheath; debility and marasmus advance rapidly, and death often occurs on or before the eighth or tenth day. The disease may, however, become chronic, and then usually becomes complicated with farcy. On dissection (Rayer, p. 737 et seq.) the *Schneiderian membrane* presents appearances varying with the intensity and duration of the disease. Sometimes it is merely occupied by solid red papulæ, which more frequently have assumed the form of true pustules, a variable number of which may further have become ulcerated, so that often a considerable extent of the membrane is destroyed. Similar appearances often exist on the *velum* and in the *larynx* and *trachea*. The lungs almost constantly present ecchymosis, and points of circumscribed lobular pneumonia, often in the stage of suppuration. When farcy coexists with pustular glanders (to which variety acute glanders in man most frequently corresponds) the subcutaneous cellular tissue of the abdomen, &c. is infiltrated with yellowish serum; and, as occurs in the human subject, abscesses often exist in the subcutaneous and intermuscular cellular tissue, and lymph, infiltrated with pus, has been found in the vicinity of the joints.

B. *Gangrenous Glanders* commences with the same general symptoms as the preceding variety. The Schneiderian membrane presents a punctated redness, which augments and spreads so that it assumes a livid aspect; there is a sanguineous nasal discharge, and the limbs, nose, and scrotum become œdematous and swollen. After two, or at the utmost three days, the pituitary membrane sloughs in patches, whence ulcers result; the discharge augments and acquires a gangrenous fetor, and death rapidly follows. On dissection the *Schneiderian membrane* is found ecchymosed, thickened, infiltrated with blood, and in various situations, especially on the septum, softened down into a putrid fetid pulp. In the *trachea* petechiæ often exist. *The lungs* are usually ecchymosed on the surface, and in four out of five cases M. Rayer observed hemorrhagic pneumonia, with purulent infiltration or gangrenous softening of the lung.

*Chronic Farcy* is characterized by subcutaneous tumours of variable shape, often forming a knotted chain in the course of the lymphatic vessels. They are at first painful, soon become hard and chronic, but finally soften and open, giving issue to a whitish, ropy, oily, characteristic discharge, and the resulting ulcers are indisposed to heal. In fact, chronic inflammation of the lymphatic system, subcutaneous abscesses, and cutaneous ulcers, are the prominent phenomena of this disease; which often terminates in, or, on the other hand, may be consequent on glanders. The post-mortem appearances have been scarcely investigated.

*Acute Farcy* seems also to consist essentially in an inflammation of the lymphatics, but it runs a more rapid course than the preceding variety. Numerous tumours form over the entire surface, and in four or five days soften and ulcerate; at this period also the appearances of acute pustular glanders manifest themselves in the nasal fossæ, and death then rapidly follows. The post-mortem appearances, so far as they are known, have been already mentioned.

*Glanders in the Human Subject.* When we recollect that perhaps not more than fifty supposed cases of this disease are on record, and that many of them are but imperfectly detailed, it is obvious that we have not yet the materials for constructing a complete history of the malady. Dr. Levin has almost confined himself to giving an abstract of all the recorded cases which he could discover; he has added but six cases to those previously collected by Rayer, only one of which, however, (Wiggin's Amer. Jour. Med. Sc., 1837,) can be admitted as an unexceptionable example of the disease; the remaining cases, one recorded by Mr. Levison, (Lancet, 1830-31, v. xi. p. 805,) and four observed by Tonelli, being at the least open to doubt. On the other hand, four of Rayer's cases have escaped his notice, as have also several others published subsequently to Rayer's Memoir, to which we shall have occasion presently to allude. We cannot of course give an analysis of the cases, but shall examine the conclusions deducible from them.

Dr. Levin admits, as did Rayer previously, that the human subject may contract glanders by infection or by inoculation, and thence he establishes two varieties of the disease: 1st, That communicated by infection, which commences with general symptoms, runs a course corresponding to acute glanders in the horse, and is uniformly fatal; 2d, That contracted by inoculation, in which local symptoms precede the constitutional disturbance, which is analogous to farcy rather than glanders, and though very fatal is less rapidly and certainly so than the preceding variety. This view seems correct to a certain extent, but requires considerable correction, inasmuch as it leaves out of consideration the form of disease under which the animal whence the disease has been derived labours; a circumstance which there is little doubt exerts an important influence on the communicated malady; and also limits the affection in man to two varieties, whereas it may probably assume in the human subject four distinct forms. We shall consequently, in considering glanders in the human subject, correct and extend Dr. Levin's views by a reference to Rayer's Memoir, in which these distinctions are fully discussed. And thus, instead of admitting but two varieties of the disease depending on the *mode of transmission*, we shall have to examine whether the four forms of the disease to which the horse is liable are capable of producing corresponding affections in man.

*Acute Glanders in Man* may, according to Rayer, assume, as in the solipedes, the pustular or gangrenous form of the disease; we do not deny that this may ultimately prove to be true; but the facts hitherto known do not seem to justify the assumption.

Acute glanders commences with general or local symptoms, being in the former case probably communicated by general infection, in the latter by inoculation. The mode of invasion, character, and order of succession of the symptoms varies, probably considerably, in different cases, and cannot yet be determined with precision, both because of the fewness of the cases hitherto observed, but more especially because, in most instances, the description of the early symptoms is imperfect, the patients not having come under medical observation until the disease had made some progress, or was even drawing to its termination. There is at first fever of variable character; general uneasiness, rigors, headach, thirst, may all or any of them be present. In some cases the fever seems of a typhoid character from the commencement; in very many instances it assumes all the characters of rheumatic fever, and has been treated as such. (A. Brown, London Med. Gaz., June, 1837, p. 353. Husson, Bull. de l'Acad. Royal. de Méd., 15 No., 1839, p. 119. Berard, meme ouvr., 15 Mars, 1840, p. 601.) In one case the initial symptoms were those of acute angina tonsillaris, (Macdonnell, London Med. Gaz., v. xix. p. 939.) Sometimes, but rarely, the disease commences with gastric symptoms, as nausea, vomiting, or diarrhœa; (Remer, Hufeland's Journ., Bd. 410, p. 58.) Whatever the symptoms of invasion may be, they are in the great majority of cases followed by pains in the limbs and joints, which have very commonly caused the case to be regarded as one of rheumatism. In some instances but a few days elapse before local symptoms supervene. In others, e. g., that recorded by Schilling, (Rust's Magazine, Bd. xi. p. 480,) the patient was affected with catarrhal, rheumatic, and abdominal symptoms, for *upwards of six weeks* before the next order of symptoms set in.

After a variable period, then, hard circumscribed subcutaneous tumours form on the parts that are the seat of pain, in the vicinity of the joints or elsewhere on the extremities, or on the trunk. The skin covering the tumours may fall into gangrene, but they usually suppurate, and when opened generally yield a sanious or bloody discharge. Between the fourth and the sixteenth days a nasal discharge appears, not however uniformly, as it was absent in four of the fifteen cases collected by Rayer. It is right however to observe that, sometimes at least, this symptom is only apparently absent; pressure in some cases causing a discharge from the nose, while in others the dorsal decubitus causes it to run into the mouth. The discharge is usually from both nostrils, is rarely abundant, is yellowish, viscid, and sometimes purulent and streaked with blood. The nose and adjacent parts are occasionally swollen, and in two cases at least gangrene of the nose occurred. At an uncertain period of the malady, at a mean term, perhaps on the twelfth day, a principal and remarkable symptom occurs, which consists in the appearance of a pustular eruption or gangrenous bullæ on the face, trunk, extremities, or genital organs. The pustules appear in succession and usually occupy the face, arms, thighs, and anterior surface of the trunk ; they have been compared to the pustules of smallpox, &c., but their appearance is peculiar and specific. The bullæ may be followed by gangrene, varying in extent and depth. Whatever the original type of the fever may have been, it now becomes of a typhoid or adynamic character. The duration of the disease is short; in two thirds of the cases death occurred before the seventeenth day; one only survived on the fifty-ninth day, but in this instance acute glanders supervened on farcy. Every case hitherto observed has proved fatal, with one exception, (Hertneig, Rayer, op. cit., p. 703,) and this case scarcely seems to come within the category of acute glanders.

When acute glanders is contracted by inoculation, from two to eight days elapse before any remarkable symptoms arise. The wound then becomes painful and swollen, and pain and inflammation extend in the direction of the lymphatics, whose course is marked by a knotted cord, or by streaked redness of the skin, the neighbouring lymphatic glands become engaged, and diffuse inflammation of the cellular tissue may occur; in a word, the symptoms resemble those produced by the inoculation of various morbid animal products; sometimes the local symptoms have been mild, and even subsided, before the symptoms above described as those of acute glanders appeared; and it is the supervention of these symptoms on the local derangement which distinguishes the case from other kinds of infected wounds.

The *post-mortem appearances* have, in most cases, been imperfectly examined. The external lesions are sufficiently indicated in the preceding account of the disease. In the cases collected by Rayer, the nasal fossæ were examined only four times, but this deficiency has been since supplied in several cases which may be found in the Bull. de l'Acad. Royale de Méd., for the years 1838-39-40. In all these cases characteristic lesions were found in the nasal fossæ; consisting sometimes in ecchymosis and gangrene, but more frequently in a peculiar pustular eruption or ulceration, or a granular condition of the Schneiderian membrane. The *larynx* in all the cases in which it was examined exhibited a pustular eruption, or ulcers, or else was inflamed and of a livid colour. Similar appearances have been detected, but not constantly, in the trachea. The *lungs* in a few instances appeared healthy, but usually exhibited lobular pneumonia, pleurisy, pleuro-pneumonia, or great sanguineous congestion.

*Chronic Glanders in Man.* Dr. Levin does not allude to this form of the disease, which we shall however briefly notice. Rayer could discover but three cases which seemed referrible to the chronic glanders; one published by Mr. Travers, the others by Mr. Hardwicke, (British Annals of Med., No. vii. Feb. 17, 1837, p. 196.) In all these cases symptoms of chronic farcy preceded those of glanders. A fourth has however been since recorded by M. Laugier, (Bull. de l'Acad. etc., 15 Sept. 1839, p. 1037,) which is a more decided and unexceptionable example of the disease. This, like the preceding variety, can probably be communicated both by infection and inoculation; at least in three of the four cases above mentioned no local symptoms of inoculation could be detected. In the case transmitted by inoculation there was inflammation of the lymphatic vessels and glands, and six months subsequently a nasal discharge with ulceration of the Schneiderian membrane appeared. In a second case the symptoms of glanders occurred after the patient had laboured for four months under pains in the joints, inflamed patches on the extremities, and subcutaneous abscesses. In a third case symptoms of farcy existed for fourteen months before the supervention of glanders. In M. Laugier's case, the patient, a negro, was first treated for rheumatism, numerous abscesses formed on the extremities, and under the use of tonics matters improved so much that he was deemed convalescent, but died suddenly in the sixth month of the disease. There had *never been any pain in or discharge from the nose*; but on dissection, the Schneiderian membrane was found thickened, of a dusky red colour, and extensively ulcerated, and the vomer necrosed and perforated. The nasal fossæ were not examined in the other three cases, in one of which ulcers were found in the trachea, larynx, and epiglottis, and small abscesses in the lungs, especially on their surface.

Dr. Elliotson (Med. Chir. Tr. 1835, v. xix., p. 237,) speaks of having cured two cases of chronic glanders in the human subject by the introduction of a dilute solution of creosote into the nostrils, and expresses his intention of publishing the cases, as he "never read of or met with any instance like them in the human subject, former cases having been acute glanders or chronic farcy:" the cases have not however, so far as we know, been yet published; and with reference to the observation just quoted we may remark that Dr. Elliotson, in his previous paper (Op. cit. 1830, vol. xvi.) calls *both* Mr. Travers's cases "*chronic glanders*," (p. 195); and in p. 199 speaks of the "second case of *chronic glanders*."

Of the four cases above mentioned one only (Travers's) recovered; but at the expiration of two years and a half the patient despaired of ever regaining his pristine vigour. An ass was inoculated by Mr. Sewell with pus from the ulcers of this patient, and died glandered.

*Acute Farcy.* Dr. Levin considers it doubtful whether farcy has ever been transmitted to the human subject, inasmuch as when infection has been derived from a farcied horse, the animal may also have laboured under glanders; but this objection is of no weight, the question being whether man is capable of contracting from the horse, whether glandered or farcied, a disease corresponding in its symptoms and lesions to farcy in the horse. And further, glanders and farcy are capable of mutually propagating each other, and some cases in the human subject which it would be difficult not to recognize as farcy, have been contracted from horses labouring under glanders, (Eck, in Rayer, op. cit., p. 774.)

*Acute Farcy* can be transmitted by inoculation, and possibly by infection; when resulting from a wound, the initial symptoms are those of inflammation of the lymphatic vessels and glands, and diffuse inflammation of the cellular tissue; and subcutaneous abscesses soon follow. These symptoms are accompanied with fever, rigors, loss of appetite, &c.; so far the case resembles some other morbid inoculations, certain dissection wounds for example, and the progress of the malady may be here arrested, and recovery ensue: if however the system becomes infected, two important sets of symptoms follow: 1st, a gangrenous and pustular eruption, similar to that which occurs in acute glanders; 2dly, numerous soft doughy tumours form on various parts of the surface, remote from the point of inoculation usually on the extremities; these tumours are seldom resolved, but almost constantly suppurate, and sometimes pass into gangrene; large subcutaneous abscesses also sometimes form on the limbs. Pains in the limbs and sometimes in the joints, always precede the formation of these tumours and abscesses; when these symptoms, especially the pustular eruptions, appear, death almost uniformly follows with symptoms of putrid fever, usually between the thirteenth and nineteenth day. When the eruption does not appear, the prognosis is more favorable, but the case may be protracted for months.

The anatomical lesions have been little examined. The nasal fossæ have not been inspected, and we only know that in addition to the subcutaneous and muscular abscesses, appearances have been found in the lungs exactly similar to those described as occurring in acute glanders.

It will be observed that acute farcy has been separated from glanders, because of the absence of a nasal discharge, and of symptoms indicating the existence of a pustular eruption, or ulceration of the Schneiderian membrane. The case already mentioned as recorded by Laugier throws much doubt, however, on the sufficiency of this distinction, as in his patient ulceration existed in the nose, though not indicated by any symptom; and had there not been a post-mortem examination, the case would, in compliance with this distinction, have been regarded as an example of farcy.

*Chronic Farcy.* It is matter of doubt whether chronic farcy has really yet occurred in man. Rayer has collected twelve cases, some or all of which may possibly be examples of the disease; but he observes that all the cases followed wounds contracted in dissecting or operating on glandered or farcied horses, that the symptoms were very analogous to those which have resulted from certain dissection wounds; and that consequently the inoculated matter may not have communicated a specific contagion. This doubt especially applies to four of the cases in which the symptoms were confined to the arm, of which one of the fingers was wounded. Some days after the injury, phlegmonous erysipelas set in and progressively extended towards the axilla, together with the reddish streaks on the skin, the subcutaneous abscesses, and the knotted cordlike swelling of the lymphatics, which have followed some dissection wounds. Recovery followed in these cases after several months, and as the test of inoculating a horse or an ass was not applied, we certainly are not justified in regarding them as cases of farcy. The remaining three cases differ from the others in this, that symptoms, such as partial inflammation of the lymphatics and the formation of abscesses, occurred in situations remote from the seat of the primary symptoms. Pains in the joints simulating rheumatism also occurred in some cases; of these three cases but one terminated fatally. We think it more than doubtful whether even these latter cases can be regarded as examples of farcy, notwithstanding that the wounds whence the disease originated were contracted during manipulations on glandered or farcied animals. M. Rayer fully admits that dissection and other wounds have occasionally though not frequently produced similar phenomena; and gives a very remarkable illustrative case which occurred in the person of M. Roux, who having wounded his finger while performing an operation for fistula in ano became affected after fifty-five hours with pain in the finger, depression, and pain in the back of the neck; on the seventh day there was violent fever; the pain extended to the front of the neck and throat, there was difficulty in swallowing, inflammation of the tongue and throat set in, and finally nine fluctuating tumours formed on the extremities. This case alone shows that the supervention of numerous abscesses can by no means be regarded as characteristic of farcy. It is however right to add that the progress of the supposed cases of chronic farcy is much slower than where abscesses of the kind in question have resulted from dissection wounds.

*Diagnosis.* A. *Acute Glanders.* The foregoing details render any lengthened consideration of this point unnecessary. The nasal discharge, the pustules or ulcers in the nose, the peculiar pustular or gangrenous eruption on the skin, distinguish it from the consequences of dissection and similar wounds, and also from *charbon* and malignant pustule. Rayer states that malignant pustule further differs from acute glanders, in that general symptoms always precede the *pathognomic characters* of acute glanders; whereas characteristic local symptoms always precede the general symptoms in malignant pustule, *even when it is contracted by infection;* a distinction, we may remark, that seems superfluous, and which at all events would not apply to *charbon*, a malady so closely allied to malignant pustule. We are not aware of any means of diagnosticating acute glanders prior to the occurrence of the external characters above mentioned as distinctive; the patients have been supposed to labour under rheumatism, gastric derangement, &c., and the progress of the malady seems alone capable of revealing its real and formidable nature.

B. *Chronic Glanders.* In all the known cases symptoms of glanders supervene on those of farcy, to which we may therefore pass.

C. *Acute Farcy.* The absence of a nasal discharge and of pustules or ulcers in the nose distinguish acute farcy from acute glanders, and the peculiar cutaneous eruption, while it marks its close connexion with that disease, distinguishes it from all others. When this eruption is absent it must be acknowledged that a reference to its exciting cause, or the inoculation of an ass or horse, can alone distinguish it from the consequences of certain dissection and some other wounds.

D. *Chronic Farcy.* We have already seen that in this form of the complaint, only a probable diagnosis can be established; the presumption as to the nature of the complaint being chiefly based on the determination of its exciting cause. The question could, however, be decided by the inoculation of an ass.

*The contagious and infectious properties of glanders*, and *the identity of the disease in the solipedes and in the human race*, may be conveniently considered together, as these questions are intimately connected in the relation of cause and effect, and as much repetition would follow from their separate discussion.

The occurrence in the human subject of a peculiar and formidable train of symptoms, not referrible to any recognized disease, naturally excited enquiry as to the origin and nature of the observed phenomena; the antecedents of the malady seemed to connect it with the transmission of glanders or farcy from the horse, and as every subsequent investigation strengthened the probability of this etiology, the transmission of glanders to man is now pretty generally received as a demonstrated fact, and a new disease is thus admitted in modern nosology. Many objections have however been urged against this conclusion, and they deserve to be attentively considered, especially as so doing will afford an opportunity of incidentally noticing perhaps every circumstance of importance connected with the disease, that has not been yet alluded to.

In the first place the transmissibility of the disease from horse to horse has been denied by some, who thence argue, that if it be not communicable between animals of the same species, à fortiori it is not so between animals of different species; the new doctrine too, it is urged, upsets the previous notions respecting the malady, which for ages has been regarded as peculiar to the solipedes. It might be replied that new truths generally explode some received errors; but it is more to the purpose to enquire what are the received doctrines respecting glanders, an enquiry to which it would be difficult to give a satisfactory answer, though recent researches have materially diminished the uncertainty connected with this matter. The fluctuations of opinion that have obtained respecting glanders within a short period are remarkable. Some thirty years since but one kind of the disease was recognized, which was universally deemed contagious and peculiar to the solipedes. The contagiousness of the disease was next questioned by some and completely denied by many. The falsity of this doctrine becoming manifest, two forms of the disease were admitted : 1st, a chronic form, whose contagiousness was denied by many and doubted by others; and, 2dly, an acute form, admitted to be contagious, but transmissible to the solipedes alone. Acute glanders was next said to be transmissible to man. And finally, both chronic and acute glanders are averred to be each both contagious and infectious, and communicable not only to man but to numerous other animals. The balance of evidence certainly seems to favour, we would say to demonstrate, this latter opinion.

The denial of the contagiousness of glanders seems to have in a great measure arisen from overlooking two of the laws of morbid poisons: 1st, that many individuals are unsusceptible of their influence in the absence at least of peculiar predisposing causes; 2dly, that there is a period of inoculation during which the poison lies latent, manifesting no action on the system. Thus M. Bouley (Bull. de l'Acad. de Méd. 31 Oct. 1838,) argued against the contagiousness of glanders, on the grounds that of 105 healthy horses, placed in the same inclosure with forty glandered horses, seven only became infected; but it may be answered, that one in fifteen having contracted the disease, the experiment rather supports than disproves its contagiousness, the immunity of the animals that escape only illustrating the first of the foregoing laws. Again Mr. Youatt, (Lancet, 1831-32, v. xxi. p. 492,) though not denying the contagiousness of glanders, supposes that it cannot be transmitted by contact with the Schneiderian membrane, because Mr. White placed matter from a glandered horse in the nostrils of a sound one for three successive days without communicating the disease. But the experiment proves nothing, as it was not sufficiently prolonged, the fact of there being a period of incubation being overlooked. We need not however dwell longer on this point; the contagiousness of glanders is now established beyond a doubt. It has been communicated from horse to horse, from the glandered matter being swallowed, (Youatt, op. cit.;) by transfusion of the blood, (Coleman, in Travers's Enquiry concerning Constitutional Irritation, &c. 4to, 2d ed. p. 399;) by insertion under the skin, the disease then appearing after a period varying from three days to three months, (Procés Verb. de l'Ecole Vet. de Lyons, May, 1811.) Many have produced the disease by smearing the nostrils of healthy horses with the discharge from a diseased one, the disease in such cases appearing between the seventh and the fortieth day. Renault (Bull. de l'Acad. Roy. de Méd.) has transmitted the disease by injecting the matter of glanders into the veins of horses; and finally, what is important, M. Leblanc has proved, too, not merely the contagiousness of glanders, but its identity with farcy, the matter of each being respectively capable of producing the other form of the disease. The supposition that glanders is peculiar to the solipedes has also been disproved. So far as we know, indeed, they are the only animals in whom it can originate or be spontaneously generated; but M. Renault (Bull. de l'Acad. Roy. de Méd., 31 Dec. 1839, et 15 Fevr. 1840,) has in two instances produced in the sheep by inoculation both the symptoms and characteristic lesions of the disease. M. Hammant, veterinary surgeon to the Pacha of Egypt, is also said to have communicated the disease to a lion and to dogs, (Bull., 15 Aug. 1839.)

It has been also objected that the symptoms of the disease supposed to be glanders in man are not precisely similar to those manifested in the horse, and that consequently the diseases are not identical. How far this objection is valid the reader can judge by contrasting the accounts we have given of the symptoms and lesions in the horse and in man. We may here refer to Dr. Levin's parallel of the disease in the horse and in the human subject, again observing that he does not distinguish the different forms which the disease is capable of assuming: 1st, *a nasal discharge*, constant in the horse, is often absent in man; but we have already shown that this absence is sometimes only apparent, and further, that ulceration may exist in the nose without this symptom being present; 2d, *the lesions in the nasal fossæ*, so constant in the horse, have been detected in the human subject whenever a suitable examination was made; 3d, *tumefaction of the submaxillary glands* has not been observed in man, but perhaps finds its analogue in the tumefaction of other lymphatic glands; 4th, *abscesses in the cellular tissue* are common to the horse and to man; 5th, *the pustular eruption on the skin* so common in man finds no counterpart, Dr. Levin states, in the horse; but this is a mistake; it sometimes does exist, and has been observed by MM. Dupuy, Leblanc, and Delafond, (Bull. de l'Acad. de Méd., 15 Nov. 1839, p. 131;) 6th, *the lesions in the lungs* are very similar in the man and in the horse. We may add that the symptoms, though thus very similar, are certainly not identical; and it would be indeed strange if they were, for it is well known that a morbid poison will often, even in animals of the same species, produce very dissimilar effects; we need only in illustration hint at the variable effects of scarlatina on the throat and skin, and the multiform phenomena of syphilis in different individuals. Mr. Coleman (Travers, op. cit. p. 400,) denies the transmission of glanders to the human subject, because of the want of identity in the symptoms, and yet in the very same passage he affords abundant refutation of his argument by stating, when speaking of glanders and farcy in the horse and the ass, "The matter of both diseases in quality is the same; we can procure glanders from the farcy matter, and farcy from glandered matter." If, then, one and the same disease produces such varied symptoms in one and the same species of animal, why because of much smaller differences deny the identity of the disease when transmitted to *another* animal species, in which a much wider range of variation might be reasonably expected?

The last objection perhaps that we need notice is, that were glanders transmissible to man, the disease should have been long since and much more frequently observed. To this it has been replied, that the disease passed unnoticed, because its transmissibility was not suspected, and thence the cases when they did occur were wrongly interpreted. That glanders has thus occurred and been overlooked in the human subject is, to say the least, very probable. Thus Rayer cites five cases which there is reason to believe were of this nature, and which have been recorded as examples of rare and unknown forms of disease; it is remarkable that of these five patients, one was a veterinary pupil, another a groom, and a third was in the horse artillery; these cases may be found in Rayer's Memoir, (p. 803 et seq.) Dr. Elliotson too says, "Since the publication of my first paper, upwards of a dozen cases have been communicated to me by medical men, which they are now satisfied were instances of glanders," (Med. Chir. Tr. 1833, v. xviii. p. 201.) Dr. Graves also says, (London Med. Gaz. v. xix. p. 939,) "From the notices which I have been able to collect, it appears that glanders in man is of very frequent occurrence in Ireland:" the particulars of the information thus alluded to are not detailed, which in a matter of such vast interest and importance is to be regretted. The tendency to the cyclical development of particular diseases may be just hinted at as possibly bearing on the subject.

We shall now, without entering into detail, notice the arguments in favour of the existence of glanders in man, and they may be thus briefly stated: 1st, A number of patients have been affected with a peculiar disease previously undescribed, and marked by certain determinate symptoms; 2d, all the persons thus affected have been in intimate relation with glandered horses; 3d, the symptoms during life, and lesions after death, correspond to those of glanders in the horse; and 4th, in several instances, matter from abscesses of the human subject produced, by inoculation, glanders in the horse and ass, (Travers, op. cit. p. 397;

Youatt, Lancet, loc. cit.; Nouat et Boulley, Bull. de l'Acad. &c. Mai, 1839.) We may here observe, that we need not wonder at the transmission of glanders to man having been long overlooked, when we find that Mr. Travers, Mr. Coleman, and Mr. Youatt refused to admit the possibility of the occurrence, after the fact had been demonstrated by the retransmission of the disease from man to the horse or ass.

As to the *mode of transmission* of the disease to the human subject, we need only remark that it certainly can be communicated by inoculation, probably by the simple contact of glandered matter with the unbroken skin; and that there are strong grounds for admitting, provisionally at least, that it can also be communicated by general infection merely, and without any local contact. The period of incubation in the human subject has not been determined. What the susceptibility of the human race to contract the disease may be it is impossible to determine, but comparing the rarity of its occurrence with the great frequency of the opportunities for its transmission, we must suppose that most individuals are but little susceptible of its influence. The predisposing causes are unknown further than this, that the majority of persons affected, especially with acute glanders, were in ill health, or of dissipated habits.

There are at least two cases which tend to show that the disease can be communicated from man to man. One will be found in the Lancet, 11th Feb. 1832. The second is mentioned by Dr. R. Williams, (on Morbid Poisons, vol. ii. p. 372.)

The *prophylactic measures* to be adopted by those placed in circumstances favouring the transmission of the disease are so obvious, that we need not occupy space by detailing them. As to the *treatment*, we find nothing in Dr. Levin's work requiring notice; and we need only say, that in the acute form of the disease, every plan of treatment hitherto adopted has proved inefficacious; while in the chronic variety the disease seemed to run a certain course, uninfluenced by remedial measures.

II. Malignant Carbuncle. The German word *milzbrand* is a popular expression corresponding to the French terms *charbon*, *fièvre charbonneuse*, *pustule maligne*, &c. under which names several different gangrenous diseases were long confounded: their signification is now much more precise than formerly, though yet perhaps somewhat lax; as it may be questioned whether the diseases thus denoted are distinct though allied, or are merely various forms of one common malady. Dr. Levin groups the several diseases or forms of disease in question under the common name of *typhus carbuncularis*, and regards them, we think correctly, as but varieties of the same affection.

The *morbus carbuncularis* may originate spontaneously in almost every domestic animal, especially black cattle, sheep, and goats; horses, asses, mules, and dogs frequently suffer; swine are more rarely affected, and even fowl are occasionally attacked. It is certainly capable of being transmitted to man; whether it can originate spontaneously in the human subject is at least doubtful.

Dr. Levin admits the following forms of the disease: 1st, *typhus carbuncularis acutissimus;* and 2d, *typhus carbuncularis acutus*, which latter form admits of the following subdivisions: a, *essential anthrax;*

B, *glossanthrax;* C, *stomanthrax;* D, *angina carbuncularis;* E, *erysipelas epizooticum.*

From a very attentive consideration of what has been written on the subject, we think that the *morbus carbuncularis*, the *maladie charbonneuse* of the French, may occur in three forms: 1st, as a general affection of a low or adynamic type without any local symptoms; 2d, as a general affection on which local symptoms supervene; 3d, as a local disease followed by general symptoms. This simpler arrangement will enable us to analyse Dr. Levin's observations more succinctly than could be done by adhering to his divisions.

The first form of the disease runs so rapid a course, that there is not time for the development of local symptoms, which otherwise we may presume would appear. This form corresponds to the *typhus carb. acutissimus* of Dr. Levin. Its progress is surprisingly rapid, varying from twenty or thirty minutes to a few hours. Sometimes indeed the animal dies almost suddenly, but more usually appears oppressed, restless, tosses the head and staggers, at one moment stands stupefied and motionless, then becomes excited, bellows, and runs about apparently infuriated; at length convulsions occur, the pulsation of the heart ceases, and death supervenes accompanied with bleeding from the mouth, nose, and rectum. After death the abdomen soon swells, and the body passes rapidly into putrefaction. On dissection dark ecchymosed or anthraceous tumours are found in the spleen, pancreas, and liver, and ecchymoses or extravasations of dark blood exist in the brain, on the surface and in the substance of the lungs, in the intestines, the cellular tissue and the muscles.

The second form of the disease, when local symptoms supervene on the general ones, corresponds to Dr. Levin's *Typhus carb. acutus* in one at least of its subdivisions and to the *charbon symptomatique* of Chabert, (Observ. sur les Malad. des Anim. dom., Paris, 1812;) it is more common than the former in black cattle and horses. The animal suddenly gets dispirited and shivers, and after the lapse of from six to thirty-six hours, anthraceous tumours similar to those to be presently described, make their appearance on various parts of the surface; they usually run rapidly into gangrene, and the animal dies in the utmost exhaustion. On dissection, gangrenous tumours or sphacelated patches are found in the principal viscera, as in the former case. The *angina carbuncularis* of Dr. Levin seems to be but a form of this disease, in which the anthraceous tumour forms in the throat or fauces, and occurs more frequently perhaps in swine than in other animals.

The third form of the disease, where the local precede the general symptoms, includes the *essential anthrax* of Dr. Levin, the *charbon essentiel* of M. Chabert, (op. cit.) It begins in the horse as a hard tumour, about the size of a bean, adherent at the base, very painful on pressure, and often presenting a minute opening on the surface; in horned cattle the tumour is larger, less painful, and rarely presents any opening. These tumours may occur on any part of the surface, but more especially whenever there is much lax cellular tissue. As the tumour increases in size, symptoms of inflammatory fever occur; but they rapidly run into gangrene, and when they do so, the fever becomes adynamic, and the animal sinks, usually, or at least often, dying convulsed. The *charbon essentiel* presents many varieties: sometimes it augments in size with astonishing rapidity; occasionally, it has been said, increasing from the size of a nut to the bulk of a man's head within an hour (?) and rapidly extending throughout the entire cellular tissue, so that the animal becomes stiff, rigid, unable to move, and dies when the tumefaction reaches the throat. The disease again may commence by phlyctenæ or hard kernels on the tongue, which soon run into gangrenous ulcers; this variety is termed *glossanthrax*, and is rapidly fatal: Dr. Levin's *stomanthrax* is merely a variety of *glossanthrax*, which occurs in swine principally. Other varieties of the disease exist on which we need not dwell. Thus in swine more particularly, but sometimes in the cow and sheep, it may commence by white spots which soon become livid and black. On dissection, the blood is found to be very dark and fluid, and sometimes exhibiting a kind of putrescence. Leuret (Archiv. gen. de Méd., t. x., p. 78,) has particularly described the physical and chemical alterations of the blood in this disease, and his statements have been confirmed by M. Delafond, (Bull. de l'Acad. Roy. de Méd., 31 Janv., 1839, p. 329.)

Dr. Levin does not mention the form of the disease called *malignant pustule* as affecting cattle, and we must confess that it is difficult to draw a line of distinction between it and the *charbon essentiel*. The *malignant pustule* (Hartnel d'Arboval, Dict. de Méd. Vet., t. iii. pp. 711-14,) is considered to differ from *charbon*, in never being spontaneous, but always originating from contact with, or inoculation by, some fluid or tissue of an animal labouring under charbon. It commences as a pustule or vesicle seated on a hard base, which extends rapidly, the base at the same time enlarging and becoming surrounded by a circular areola of variable colour, and covered with phlyctenæ full of reddish serum; this areola soon becomes prominent, so that the centre of the tumour, which resembles a hard eschar, seems depressed; the cellular membrane rapidly passes into gangrene, and the parts surrounding the gangrened structures become so remarkably tense and swollen as to give quite a characteristic aspect to the affection. As the disease advances, constitutional symptoms set in, and death soon occurs.

Much has been written, but little is known, respecting the causes of these carbuncular diseases. The affection, so far as we know, is extremely unfrequent in these countries; it seems to be more common in certain districts of France than elsewhere; in Franche-Comté, Burgundy, Lorraine, the Basses-Alpes, and Languedoc, for example, it appears frequently and epidemically. Its origin has been attributed to extreme wet, or on the other hand to extreme drought, or long-continued heat; to imperfect nourishment, or to the prevalence of unwholesome plants (especially the ranunculaceæ?) in the pastures. Whatever the original cause may be, it is demonstrated that the disease when once in existence can be propagated by contagion, as, independent of popular experience, M. Leuret (loc. cit.) has proved by direct experiment, that not only will the matter of the gangrenous tumours, but the blood or portions of the lung or liver of an affected animal, produce the disease if inserted in the cellular tissue of a healthy one.

*Malignant Carbuncle and Pustule in the Human Subject.* Dr. Levin remarks that the *morbus carbuncularis*, as it affects man, was known long before its exciting cause was suspected, and justly observes that great confusion exists in its description as given by authors. He simplifies the matter by describing but one of the forms of the disease that occur in man, as his observations relate to the *malignant pustule* alone.

The truth is that the authorities differ extremely as to the distinctions which should be established between the various diseases long confounded under the common appellation of *charbon*. It would be useless to recount the various opinions which have been put forward on this subject, and we shall content ourselves with saying that a careful review of the facts seems to bear out the opinion of MM. Marjolin and Oliver, (Dict. de Méd. 2de ed., 1834, t. vii. p. 272,) who admit two varieties of the disease: 1st, malignant charbon, which we shall call anthrax or carbuncle; and 2dly, malignant pustule.

*Malignant Anthrax.* This disease is the most formidable of all external tumours; it is almost uniformly preceded by depression and by prostration of strength, which is often not perceived till the patient makes some exertion; nausea, cardialgia, and syncope sometimes exist. The tumour, which may form on any part of the body, almost constantly appears on the face, chest, neck, or upper extremities; it is not very prominent, is very hard and painful, of a dark or livid colour in the centre, and shining red towards the circumference; it is almost always preceded or accompanied by one or more dark pustules or small livid vesicles, which soon open and yield a reddish irritating discharge, which causes intolerable heat and itching; the base of the tumour is surrounded by an inflamed areola, varying in colour and extent according to the intensity of the disease. The centre of the tumour soon becomes a hard, dry, black eschar, or a soft brown slough; burning heat and pain, and a very distressing sense of constriction are invariably felt in the part. Gangrene extends from the centre to the circumference, and often destroys a great extent of the skin, cellular tissue, and even the muscles; intense fever sets in early, the pulse is either small and contracted, or on the contrary tolerably full; the skin is usually dry, but intermittent sweats have been observed. Thirst is often intense, but sometimes is absent; precordial pain and anxiety is almost constantly complained of; and typhoid symptoms soon set in, under which the patient sinks.

The progress of the disease is sometimes astonishingly rapid: Fournier has called it *une tumeur de surprise;* death has occurred within twenty-four, nay even ten, hours. As the occasionally extraordinary rapidity of this disease is not dwelt on in most of the books, we shall notice a case recorded by M. Vidal de Cassis, (Traité de Path. ext., t. i. p. 191.) A man was admitted to the Hôtel Dieu, Marseilles, at 1 p. m., with a small, hard, brown, extremely painful tumour on the neck; at 3 p. m. the neck was so enormously swollen, as to be on a level with the face and thorax; large phlyctenæ seated on hard black bases had formed on the skin, and death occurred at 6 p. m., within five hours after his admission with an apparently insignificant ailment. When the disease affects the face or trunk, the prognosis is much more unfavorable than when it occupies the extremities; but if situated over a large artery or joint, the opening of the vessel or articulation is to be apprehended. Ambrose Paré (Œuvres, liv. xxiii.) saw one of these tumours occupying the abdomen denude the peritoneum to the extent of a hand's breadth.

*Post-mortem Appearances.* The nature of the local lesions can be sufficiently understood from the description of the disease. The heart is usually softened, and presents numerous ecchymoses, especially on the internal surface of the cavities, and on the left side; the blood is often liquid and black, particularly in the veins; numerous ecchymoses exist in the lungs, on the external and internal surface of the intestines; and the liver and spleen are gorged with blood and easily broken down.

The prognosis is most unfavorable, death occurring almost constantly and rapidly. Such terror indeed did the disease formerly inspire that the patients were secluded and abandoned without help. (Fournier, Observ. et Expér. sur le Charbon mal., 1768, p. 9.)

*Malignant Pustule.* This is the form of the disease to which Dr. Levin exclusively refers, and his account of it is neither very clear nor precise. As a description of this disease would occupy much space, and especially as an admirable account of it may be found in so accessible a work as Boyer's Surgery, we shall not notice its symptoms, but merely mention the characters which distinguish it from *malignant charbon* or *anthrax*, a point on which Dr. Levin of course does not touch, as he does not mention this latter affection.

The two forms of disease, which we now seek to discriminate, were long confounded, and it is certainly difficult to draw a strict line of demarcation between them; they can by no means be regarded as essentially distinct, being clearly but varieties of the carbuncular malady; but yet they present differences which forbid us to regard them as identical. The first distinction between them is that in *charbon*, general symptoms precede the local symptoms, the reverse being the case in *malignant pustule*. *Charbon* runs a more rapid course, is attended with more decided and serious adynamic symptoms, and is much more constantly fatal than malignant pustule. The external characters of the two affections also differ. In *malignant pustule* there is around the gangrened part an enormous tumefaction of the cellular tissue of a kind which does not occur in charbon; the appearance of this swelling is said to convey the idea that it is emphysematous; the skin covering it is tense and paler than natural, but it does not crepitate, being usually exceedingly hard, but sometimes soft and elastic. Charbon is also much more painful than malignant pustule. Whether the etiology of these affectious serves further to distinguish them we shall presently examine.

Dr. Levin devotes a chapter to the consideration of *the transmissibility of the carbuncular disease from the lower animals to man*, and observes that we must first determine whether it is transmissible amongst the lower animals themselves. This enquiry, he remarks, is embarrassed by the disease occurring as an epizootic, which allows the facts calculated to establish its infectious and contagious character to be plausibly explained by a reference to the prevailing epidemic influence. The results of experiments on the subject have not been uniform, but since the publication of Leuret's Memoir (Loc. cit.), with which Dr. Levin does not seem to be acquainted, no reasonable doubt can remain that the disease can be propagated by contagion. Whether it is infectious is still an open question.

Dr. Levin considers it as undetermined whether the carbuncular disease can be communicated to the human subject by infection, but refers to no authorities on this head. We have met with but one definite statement bearing on the point. Chaussier (Méthode, etc. suivi d'un Précis sur la Pust. malig., p. 117,) says an individual contracted charbon from the emanations from the fæces of a patient labouring under gangrenous fever; but the fact is deficient in precision. Whatever may be the state of the case as regards charbon, it seems universally admitted that malignant pustule is never the result of infection. Dr. Levin admits that the disease is transmissible to man by contagion or inoculation; and we shall advert to the chief grounds on which this opinion rests.

The persons most commonly affected with this disease are butchers, tanners, and others whose occupation brings them into contact with animals or animal remains; and the parts of the body which are usually uncovered (as the face, neck, chest, arms, hands, and in certain trades the feet,) are almost exclusively affected; the disease, however, does appear in other situations, but always under such circumstances that the exceptions strengthen the argument: thus a butcher while slaughtering a diseased beast placed his knife between his teeth and the malady appeared in the mouth; the blood of a slaughtered animal trickled down the back of a man who was carrying it and the disease broke out on the parts with which the blood had come in contact. Again, numerous examples are on record of the direct transmission of the disease to the human subject, some of which are so curious, that though familiarly known we may be pardoned for alluding to them. Thus Bertrandi (Oper. Chirur. t. i. p. 100) saw two fatal cases which originated from the contact of a fly that had fed on the body of an animal that died while labouring under charbon; and Monteggia (Instituz. Chir., t. i. p. 197) gives a similar case. The disease has followed the introduction of the arm into the fundament of a horse; but we need not multiply instances, numbers of which are recorded by Chaussier, Fournier, and Thomassin.

Dr. Levin states that Gasparin has observed the disease arise from contact with the recent blood of an affected animal, while the flesh when thoroughly blooded was innocuous; and that, according to Hoffman, the contact is most dangerous while the animal is alive, and the more so the shorter the period that has elapsed since death. An important question is whether the disease can be communicated by eating the flesh of animals affected with it; this point Dr. Levin considers very fully and quotes a number of contradictory authorities. Thus Morand (Oper. de Chir., t. i., p. 236) gives a case of two butchers who killed an over-driven but apparently healthy ox and became affected with charbon, while many persons ate of the flesh with impunity. Again, a butcher, his wife, and assistant, all contracted charbon from an animal labouring under the disease, but upwards of 100 individuals ate of the flesh without sustaining any inconvenience. Guillo de Besançon, Camerarius, and Gerberius say that the flesh of animals that died of the malady has been frequently supplied to soldiers without any bad result. On the other hand Chaussier gives cases in which the most terrible consequences followed the use of such meat. Some have endeavoured to reconcile these contradictory facts by referring the mischief to the meat having been imperfectly cooked, (Hanke, Neue Breslauer Sam. aus dem Gebiet der Heilkunst, 1829, Bd. i., p. 564; Morand, op. cit.) But A. Ammon (Unterricht über den Milzbrand, p. 60,) gives cases where death quickly followed after eating the flesh of animals affected with the disease, though it was thoroughly cooked. Gasparin conjectures that the flesh of animals who die in an early stage of the malady is innocuous, while it is prejudicial if the disease has existed for a certain period; he also attaches much importance to the flesh being thoroughly blooded, which he thinks calculated to remove its prejudicial qualities. Amidst all this uncertainty Dr. Levin justly remarks that the flesh of animals that die of this disease should not be employed as food.

It is interesting to determine whether charbon is transmissible from one human being to another. As examples of such transmission Dr. Levin quotesa case from Thomassin, (Diss. sur le Charb. malig. etc., p. 31,) where a woman contracted the disease by contact from her husband, and also a case recorded by Helbich, (Diss. Inaug. de Carb. malig., Berol. 1827, p. 16,) in which a child communicated the malady to its mother. He also refers to other cases by Barez (Hufeland's Jour. Dec. 1822, p. 95,) and Hausbrand (Medicin. Vereinszeitung, 1836, No. xxix, p. 145); but does not mention two very conclusive cases given by Fournier (op. cit.) Notwithstanding these and other similar cases some authors, Dr. Levin observes, maintain that the disease is exhausted in man, and cannot be communicated from one human being to another, (Schönlein, Allgemein. und speciel. Path. und Therap., 3te Aufl., bd. i. p. 389.) Some experiments made by inoculation from man, to the human being accidentally and to animals designedly, favour this view, (Basedow, Graefe und Walter's Jour., 1828, p. 591; Mandt, the same, 1825, p. 201;) but we cannot admit that a few negative facts disprove the positive results observed in other cases.

Dr. Levin, in his *comparison of the disease as it occurs in man and the lower animals*, recognizes the general similarity of the symptoms and post-mortem appearances. The chief difference he considers to exist in the same relation of the general and local symptoms. The local symptoms, he says, being in the lower animals preceded by general symptoms, while in man precisely the contrary occurs. . . . . The character of the local symptoms are also, he states, completely different, consisting in the lower animals of a *hard anthraceous tumour*, and in man of a *vesicle or pustule rapidly running into spreading gangrene:* whence, he says, the term *carbunculus* as applied to the human subject should be abandoned, and the name *malignant pustule* alone retained. But we have already stated that Dr. Levin recognizes one form only of the disease in man, namely *malignant pustule*, having overlooked the carbuncular variety (*charbon*) of the malady; while on the other hand he has neglected *malignant pustule* as it occurs in brutes. The foregoing distinctions are therefore inaccurate, as the reader may readily collect from what has been stated when considering the history of the various forms of the disease.

It has been considered by some as an inexplicable amomaly that one and the same deleterious matter should be capable of producing the two forms of disease known as *charbon* and *malignant pustule*. But, as we have already remarked when speaking of glanders and farcy, such a fact is not without a parallel in the history of morbid poisons.

The chapters on the *prophylaxis* and *treatment* of the disease whether in man or in brutes call for no remark. The prophylactic measures will readily suggest themselves; and the account of the treatment of *malignant pustule* in man, to which alone Dr. Levin adverts, is but an imperfect abstract of that recommended by Boyer.

III. Aphtha Epizootica (*Maul-und Klauenseuche*). Dr. Levin observes that, though the transmissibility of this disease to the human subject seems sufficiently proved, the number of well-observed facts are too few to afford materials for a complete description of the malady as it occurs in man. Aphthæ occur symptomatically in many diseases of the lower animals; but the affection now under consideration is an epizootic of which aphthæ form an essential character. It seems analogous to the acute exanthemata, and affects many domestic animals, especially the cow, sheep, horse, and pig. It commences with fever and diminution of all the secretions, save that from the mouth, and often from the nose, there is a discharge, at first thin but subsequently more consistent; the lips and tongue swell, and together with the gums and fauces present numerous minute red points, which soon become white or yellowish vesicles, which break and expose aphthous ulcers, and the greater part of the mucous membrane of the mouth is often thus detached. The epithelium is gradually renewed, and the animal generally recovers in from ten to twenty days. During the course of the disease a vesicular inflammation usually occurs on the foot also.

Sagar (de Aphthis Pecorinis, Vien., 1765, p. 14,) was the first to notice the transmission of this disease to the human subject; he describes the patient as labouring under aphthæ and difficult deglutition. Brosche (die Maul-und Klauenseuche der Kinder, etc., Dresd. 1820, p. 27) and Böhmer (Medicin. Jahrbüch des Oesterreich. Staat., 1827, No. xlviii. p. 226) mention somewhat similar cases; but our best information on the subject is derived from the experiments of Hertwig (Medic. Vereinszeitung, 1834, No. 48, p. 226) who, during the prevalence of this epizootic at Berlin, along with two other physicians drank daily for four consecutive days a quart of milk obtained from cows labouring under the disease. On the second day Hertwig became affected with slight fever, pain in the limbs, headach, heat and dryness in the mouth and pandiculation. These symptoms continued in a mild form for five days, when the entire mucous membrane of the mouth, especially of the tongue, became swollen and covered with minute yellowish vesicles, which enlarging burst and exposed the epithelium softened and of a dark red colour. Chewing, swallowing, or speaking caused burning pain, and there was extreme thirst. Ten days elapsed before the excoriated surfaces healed. Simultaneously with the appearance of the vesicular eruption in the mouth, vesicles which ran a similar course formed on the hands and fingers. The other two physicians were similarly affected. When the aphthæ healed their health was completely restored.

As to the *mode of transmission of this disease*, the experiments just mentioned, together with some previous observations of Sagar to the same effect, show that the disease can be communicated by the ingestion of the milk of animals affected with it. Whether the discharge from the vesicles, or the use of the flesh of animals labouring under the malady, can propagate it, is at least doubtful. We have said enough to enable our readers to judge of the parallelism of the disease in man and the lower animals. And the subject is not of sufficient importance, in the present state of our knowledge at least, to call for a more extended notice.

IV. Cowpox. The next disease of which Dr. Levin treats is the cowpox, a subject which has been so very lately and amply noticed in this Review, that we shall not again enter on its consideration on the present occasion; the more especially as Dr. Levin adds nothing to our existing stock of information.

V. THE GREASE (*Mauke*). This is a disease which is most frequently seen in the horse. It occasionally occurs in the ass and mule; and possibly some other animals may be liable to it.

Dr. Levin commences by insisting on the familiar distinction of grease into two kinds,—one merely a local disease, the other a constitutional affection preceded by fever. This distinction, as is well known, is of great importance as connected with the question of the origin of the cowpox. Dr. Levin considers the acute grease, the second of the above varieties of the disease, as analogous to the exanthemata. It commences by fever followed, in from one to three days, by local symptoms; which usually commence in the hind feet, and subsequently extend to the anterior extremities. The heel, which is uniformly the part affected, swells; becomes hot and painful, the hair gets rough and staring, and redness of the skin is occasionally perceptible. After some days a colourless or yellowish, transparent, greasy, ill-smelling ichor exudes in drops or like an exhalation from the surface, and clots the hair together. This secretion, according to some, simply exudes from the pores of the skin, and is never secreted from vesicles or pustules; but Hartnel d'Arboval regards it as originating from the follicles at the roots of the hair. The limpid secretion gradually becomes thick, puriform, extremely fetid, and produces excoriations on the surface which terminate in fissured chaps or pustules. At a more advanced stage the discharge becomes sanious, greenish, or brown, acrid, and insupportably fetid; and the hair and even portions of the skin come away, ulcers form and may extend to the cellular tissue, and cauliflower excrescences are often thrown up from their surface. The entire foot finally becomes a deformed mass covered with ulcers and wart-like granulations, the hoof falls off, and death may at length occur from exhaustion.

It is familiarly known that Jenner attributed the origin of the cowpox to the accidental transmission of the matter of grease to the udder of the cow; that Woodville, Simmons, Pearson, Barthelemy, Houzard, and many others controverted this opinion; which was upheld by Taunton, Lupton, Laffont, and others, both parties appealing to experiments in support of their opinions; and that at length Mr. Loy found that the vaccine disease was communicable to the cow and thence to man, or to man directly without the intervention of the cow, by the inoculation with the *limpid* matter of grease; but that the experiment failed if the secretion had become puriform. The protective power and identity with cowpox of the disease thus called into existence was tested by inoculating the patients experimented on with smallpox. (Bryce, Pract. Observ. on the Inoculation of the Cowpox, &c. 2d Edit., p. 17, and Appendix, No. 1.) Dr. Levin cites a more recent and a very accurately observed fact of the direct transmission of the disease to man; recorded by Hertwig, (Medic. Vereinszeitung, 1834, No. 48; Hufeland's Journ., Sept. 1830, p. 122.) In 1830 the grease was very prevalent at Berlin, and twelve persons occupied about some diseased animals became infected. They suffered

LIBRARY OF THE

Orleans Parish Medical Society from slight fever during three or four days, and then one or more fingers became painful and swollen, and the swelling extended to the hand and forearm, or even to the axilla. On the fourth or fifth day, hard red elevations appeared on the fingers, which by the ninth or eleventh day became blueish pustules. In three cases there appeared on the back of the hand and arm an eruption of pustules exactly similar to those of the cowpox. We shall adduce another case not noticed by Dr. Levin, which much more strongly supports the doctrine advocated by the illustrious Jenner; as in Hertwig's cases it does not appear that any experiments were performed on other individuals, with matter taken from the pustules of those who contracted the disease from the horse. Husson (Dict. des Sciences Méd., Art. Eaux au Jambes, t. xi., p. 98,) mentions the case of a coachman who, while attending on a horse labouring under grease, became affected with an eruption on the wrist so exactly similar to the vaccine eruption, that Husson was induced to inoculate three children with matter taken from them; perfect vaccine vesicles formed, with matter from which a series of inoculations to many degrees of descent were performed. This case would have been still more satisfactory had the specific nature of the disease been further tested, as in Mr. Loy's cases, by vaccination or a secondary inoculation with smallpox.

VI. Scabies Ferina (*Raüde*). The itch of animals or mange affects almost all the domestic animals, especially the horse, sheep, dog, and cow. We think it needless to occupy space in describing the symptoms of a disease so familiarly known, especially as the fact of its transmissibility to man may be open to some doubt, at least it is rarely so transmitted, and when it is, excites a disease of but little importance.

Dr. Levin quotes cases from Veith, (Handbuch der Veterinärkunde, Bd. ii. p. 674;) Greve, (Archiv für Thierheilk., Bd. ii. p. 46;) Fauvet, (Revue Méd., t. x.;) and Hertwig, (Medicin. Vereinszeit., 1834, No. 48, p. 225;) where persons after contact with mangy animals became affected with some eruptions, always attended with extreme itchiness; the characters of the eruption have been in hardly any instance minutely described, but it certainly differs materially in different cases. Thus in Greve's cases the patients recovered without any treatment in six or eight weeks; while in other cases (Hartnel d'Arboval, op. cit.) it continued a year despite of all treatment. The transmitted disease, Dr. Levin states, seems to differ from the scabies peculiar to the human subject, in affecting all parts of the body indiscriminately, the head and face being often attacked, and no preference shown for the hands or arms. Rayer (Treatise on Diseases of Skin, tr. by Willis, 2d Ed. p. 1203,) mentions a case not noticed by Dr. Levin, in which the transmitted disease, according to the testimony of MM. Dumeril, Geofroy St. Hilaire, and Bosc exactly resembled that proper to the human subject, save that the vesicles were much larger and attended with more intolerable itching and some redness of the skin. In most of the known cases the disease has been contracted from the horse. It has been said to have been communicated by the dog, but this is doubtful, and it has probably never been transmitted by the sheep. As to the mode of transmission it has been uniformly by contact.

VII. HYDROPHOBIA (*Hundswuth*). Dr. Levin observes that, though this disease has been known from very remote antiquity, the efforts of physicians were long so exclusively directed towards discovering some specific for its cure, that the study of the phenomena of the disease in animals was comparatively neglected, and thence he accounts for the defective and contradictory descriptions of the malady which so long passed current. Mr. Meynell's observations, recorded by Fothergill, are perhaps the first of much accuracy that were made on the subject, but for a long period they attracted little attention, the older accounts continuing to be copied by most if not all authors. At length, Blaine in England; and in Germany, Greve, (Erfahrüng und Beobacht. über die Krank der Hausthier. im vergleich. mit den Krank des Mensch;) Waldinger, (Abhand. über die gewöhnlich. Krank der Hunde;) and especially Hertwig, (Beiträge zur näher Kennt. der Wuthkrank)—from the accurate observation of above 300 animals affected with the malady, drew a more faithful picture of the disease than had been previously depicted.

Dr. Levin considers it as in the highest degree probable that the canine race alone can be primarily affected with hydrophobia. Our readers are no doubt aware that it has been hitherto universally held that hydrophobia can also be spontaneously generated in the cat; an opinion which Dr. Levin however regards as almost completely disproved by Froriep, (Casper's Wochenschrift, 1837, No. 13, &c.) who it would appear puts forward strong grounds for believing that the cat always becomes affected in consequence of having been bitten by the dog. Dr. Levin contents himself with making the foregoing statement, but does not enter into any detail respecting the facts and arguments adduced by Froriep in support of this doctrine, and consequently we are not in a predicament to form an opinion as to its validity.

*Hydrophobia in the dog* assumes, according to Dr. Levin, two distinct forms. The first of which is characterized by augmented activity of the sensorial and locomotive functions, continued and peculiar barking, and a strong disposition to bite. The disease commences with some alteration in the peculiar habits and disposition of the animal, who, as the case may be, is more irritable, more tractable, more lively, or more sluggish than usual; or these several conditions may alternate in one and the same animal. An early symptom consists in an inclination to lick or carry in the mouth various inedible substances, especially such as are cold. The animal after a time gets restless, snaps in the air as if at flies, frequently leaves the house, but usually soon returns, and is obedient and seems attached to his master. According to Blaine, constipation constantly exists. There is usually complete loss of appetite, but the animal seems to suffer from thirst, drinking eagerly, until, as indeed usually occurs, the mouth and tongue become swollen. The eyes are red and become dull, haggard and half closed, the skin of the forehead being also wrinkled, which gives the animal a peculiar aspect. The nose, tongue, or throat now usually become swollen, and the coat becomes rough and staring. According to Hertwig, the mouth is generally very dry; but Blaine has constantly observed a flow of thin saliva. After some time the gait becomes unsteady and staggering, and finally the extremities are paralysed. The tail in this form of the disease is not drawn between the legs, and the head is carried erect, the nose being pointed upwards. A

CHARITY HOSPITAL, MEDICAL LIBRARY.

disposition to bite sooner or later invariably occurs; it is not however permanent but recurs periodically; is directed both against inanimate and animate objects; most especially against the cat, less so towards other animals, and least of all towards man. When the animal bites he does not previously bark or fly at the object of his attack, but approaches in a quiet or even friendly manner, and makes a sudden snap. The *second* form of the disease is distinguished by inactivity and depression, there is no disposition to bite, probably from the lower jaw being paralysed; nor is there any inclination for change of place manifested. The first symptoms are unusual quietness and apparent depression of spirits. The voice is peculiarly altered, as it is also in the foregoing variety, but there is much less disposition to bark. The mouth is open, the lower jaw hangs as if paralysed, and is only raised under the influence of strong excitement; there is a constant flow of slaver from the mouth. The animal either does not drink at all, or does so with difficulty, but manifests no fear of water, as it, on the contrary, willingly immerses the nose in that fluid. The tongue is almost constantly protruded from the mouth. The dissection of dogs that have perished from this disease has hitherto given only negative results.

Dr. Levin, it will be seen, states that dogs *never* manifest any fear of water, and thence, as others have done before him, he thinks that the term hydrophobia should be rejected. Whether dogs never have a fear of water we really cannot pretend to say, but that they *often* have not is mentioned by all modern authors. This fact should be popularly known, as it is vulgarly thought that it can be determined whether a dog labours under this fearful malady, by observing the influence exerted on him by the sight or contact of water.

In the cat, Dr. Levin observes, the disease generally breaks out suddenly; the animal usually springing violently, but often stealing craftily on its victim; the hair is erect, the eyes sparkle, the mouth froths, and plaintive cries are uttered. The animal indulges in the most extraordinary contortions and springs, alternating with convulsions; these attacks occur in paroxysms, one of which ends in death. The cat is, however, liable to a tranquil form of the disease, which is even more formidable, as the symptoms excite little attention.

We think it needless to follow Dr. Levin in his description of this terrible disease as it occurs in the horse, cow, sheep, goat, pig, &c.; as it seems universally admitted that these animals are incapable of transmitting the disease to man or any other animal; that disastrous privilege being limited to the canine race and to the cat.

*Hydrophobia in the human subject.* Dr. Levin agrees with most writers on this disease, that its symptoms are liable to much modification, and present great variety. He avoids entering into the consideration of these varieties, merely describing the leading features of the disease as it most usually occurs. He first notices the period of incubation, and seems to admit it may last but a few hours, or on the other hand be protracted for many years; in this differing from the almost unanimous opinion of modern authorities. Dr. Elliotson, no doubt, believes that the disease has set in within a few hours after the receipt of the bite (Principles and Prac. of Med., p. 499,) but this is certainly not the received opinion; while the extreme limit is extended by perhaps no modern authority of respectability, beyond the eighteenth or twentieth month. Dr. Levin notices the various accounts that have been given of the progress of the wound, and the condition of the resulting cicatrix, which it is unnecessary to reproduce, as they will be found in all the books. To the period of incubation succeeds the first stage of the actual malady, called by Dr. Levin, in common with several authors, the *melancholy* or *convulsive stage*. There is nothing requiring notice in our author's description of this stage of the disease, except that he describes it as uniformly ushered in by or accompanied with local symptoms in the wound or cicatrix; where as in many cases no such local symptoms occur. His description of the *hydrophobic stage* calls for no remark, except that he does not allude to an alleged peculiarity in the disease when communicated by the cat; in which case it is said that the patient seldom manifests any horror of or difficulty in drinking water. The account of the post-mortem appearances is very imperfect, perhaps designedly so, as it is known that they vary exceedingly in different cases.

Various opinions have obtained as to the *mode of transmission* of hydrophobia. As regards dogs, Mr. Meynell long since maintained that such of them as were supposed to become affected merely by general infection, were in reality bitten; and Dr. Levin informs us that Hertwig never found dogs become rabid when confined in the same locality with those labouring under the disease. With respect to the transmission of the disease to man, Dr. Levin's observations are very imperfect. He merely alludes to and rather negatives the possibility of the disease being communicated by the application of rabid saliva to the unbroken skin, but makes no reference to the cases recorded by Dr. Bardsley and others, in which the disease was apparently, at least, thus transmitted. Contrary to the generally received opinion, he admits that the disease can be communicated to man by other animals than the canine race and the cat, but does not quote the cases calculated to uphold this opinion. Dr. Levin also, without however citing a single case in point, considers it problematical whether the disease may not be communicated from man to man; whereas all experience, as regards the immunity of the attendants on the sufferers, runs the other way. We may observe that such a doctrine should on no account be broached without adducing corroborating *facts*, as it is calculated to revive those fearful prejudices which added fresh horrors to this most terrible of all maladies, prejudices which are not yet indeed completely exploded, as a few months since several individuals were tried and convicted in Ireland for smothering a relative who laboured under hydrophobia (Dublin Med. Press, 1840). Dr. Levin does not allude to the numerous experiments which tend to show that the canine race and the cat are the only animals whose saliva is capable of propagating the disease. Thus Dupuy could never transfer the disease from rabid cows or goats to animals of the same species, though they were readily affected when inoculated from the cat or dog, (Dict. des Scien. Med., Art. Rage. t. 47, p. 46.) As regards the human subject there are many instances of the attendants having been bitten by or otherwise inoculated from a hydrophobic patient, but we are not aware of any case in which the disease was thus transmitted. Dogs also have been repeatedly inoculated from patients labouring under hydrophobia, and in every instance with a negative result, save in the well-known experiment performed by Magendie and Breschet in 1813, (Dict. des Scien. Med. loc. cit.) This experiment, however, stands alone, and is open to the objection that, as hydrophobia was prevalent amongst dogs at the time, the animal may have been previously bitten, or may have spontaneously contracted the disease. We feel however bound to admit, though it may be inconsistent with the doctrine we advocate, that this experiment leaves the matter in some doubt. But even were it to be repeated with a similar result, we would be scarcely justified in thence assuming that the saliva of the hydrophobic human subject could infect man; because the dog, as it spontaneously generates the disease, may be fairly supposed to be more susceptible of contracting it; and assuming Magendie's experiment to afford an undoubted instance of the transmission of the disease from the human subject to the dog, there seems to have been a diminished activity in the contagious power of the transmitted virus, as Magendie (Journ. de Physiol. t. i. p. 42) states that the transmission of the disease was arrested in the third degree of descent, the dogs bitten by the inoculated animal contracting the disease, but not transmitting it to the dogs whom they in their turn bit. This question however obviously calls for further investigation. Another point of much interest has not been touched on by Dr. Levin, the possibility, namely, of hydrophobia being communicated to man by the saliva of a healthy dog; there is, at least, one case on record in which such an event is alleged to have occurred, (Hufeland's Journ. Dec. 1839,) but this is a matter on which our judgment must be suspended. Dr. Levin states on the authority of experiments performed by Hertwig, that hydrophobia can be communicated not only by the saliva, but by the blood. No account is given of these experiments; and we may remark that Dupuytren, Breschet, and Magendie, in vain attempted to communicate the disease by injecting the blood of animals labouring under it into the veins of healthy dogs. We could swell the catalogue of interesting and important points, which are either imperfectly noticed, or passed by in silence by Dr. Levin, but we shall only allude to one more. It might be supposed from Dr. Levin's work, that hydrophobia is sure to follow a neglected bite of a rabid dog,—at least there is no allusion to the fact that a considerable number of those bitten escape the disease; what that proportion is we have not sufficient data to determine, but some approximation might be made by collecting all the recorded cases where the numbers bitten and attacked are respectively specified. The chapters on the prophylaxis and treatment of the disease, whether in man or in the lower animals, contain nothing which calls for notice or remark.

We opened Dr. Levin's work with considerable interest, in the anticipation that it might extend the previously existing account of knowledge respecting the important class of diseases of which it treats; and as we cannot indulge in eulogy, we shall leave our readers to draw their own conclusion as to how far our anticipations were satisfied.

